# Effects of Predator-Prey Interactions on Predator Traits: Differentiation of Diets and Venoms of a Marine Snail

**DOI:** 10.3390/toxins11050299

**Published:** 2019-05-25

**Authors:** David A. Weese, Thomas F. Duda

**Affiliations:** 1 Department of Biological and Environmental Sciences, Georgia College and State University, Milledgeville, GA 31061, USA; 2 Department of Ecology and Evolutionary Biology and Museum of Zoology, University of Michigan, Ann Arbor, MI 48109, USA; 3 Smithsonian Tropical Research Institute, Balboa, Ancon, Republic of Panama

**Keywords:** conotoxin, RNA-seq, differential expression, single nucleotide polymorphism, predator-prey interactions

## Abstract

Species interactions are fundamental ecological forces that can have significant impacts on the evolutionary trajectories of species. Nonetheless, the contribution of predator-prey interactions to genetic and phenotypic divergence remains largely unknown. Predatory marine snails of the family Conidae exhibit specializations for different prey items and intraspecific variation in prey utilization patterns at geographic scales. Because cone snails utilize venom to capture prey and venom peptides are direct gene products, it is feasible to examine the evolution of genes associated with changes in resource utilization. Here, we compared feeding ecologies and venom duct transcriptomes of individuals from three populations of *Conus miliaris*, a species that exhibits geographic variation in prey utilization and dietary breadth, in order to determine the extent to which dietary differences are correlated with differences in venom composition, and if expanded niche breadth is associated with increased variation in venom composition. While populations showed little to no overlap in resource utilization, taxonomic richness of prey was greatest at Easter Island. Changes in dietary breadth were associated with differences in expression patterns and increased genetic differentiation of toxin-related genes. The Easter Island population also exhibited greater diversity of toxin-related transcripts, but did not show increased variance in expression of these transcripts. These results imply that differences in dietary breadth contribute more to the structural and regulatory differentiation of venoms than differences in diet.

## 1. Introduction

Disjunct populations of widespread species are often exposed to varying environmental conditions that can lead to population differentiation and local adaptation. Indeed, geographic mosaics of interacting species may generate different selection regimes among populations [1] that can drive the genetic and phenotypic divergence of these populations and ultimately facilitate their reproductive isolation [2,3]. Traits that operate at the interface of species interactions (e.g., those directly associated with predator-prey interactions) represent some of the primary targets of selection among populations that differ in how they interact with other species and the identity and characteristics of these other species. For example, populations of male guppies exposed to different communities of predators differ in color intensity, a feature utilized by their visual predators [4]. Additionally, experimental populations of *Brassica rapa* plants diverge in floral traits in response to exposure to pollinators that are attracted by these traits [5]. Although such examples highlight the effect of species interactions on the evolution of traits linked to these interactions, the molecular mechanisms underlying many of these phenotypic changes are largely unknown.

Given that venoms are typically comprised of direct gene products that operate directly at the interface of predation [6], venomous organisms offer tractable systems for addressing questions concerning the evolution of genes associated with traits tightly linked to predator-prey interactions. Members of the marine gastropod family Conidae (cone snails) are a hyper-diverse group of over 800 species that utilize venom to capture prey [7]. Venoms of cone snails are largely comprised of small cysteine-rich peptides (conotoxins) [8] that exhibit considerable diversity among species [8,9,10]. Conotoxin genes are encoded by many large gene families [11] and evolve under positive selection [11,12]. In addition, high rates of gene gain and gene loss contribute to divergence in the composition of conotoxin gene families among species [13]. Differences in conotoxin gene expression patterns are also associated with interspecific differences in venom composition [10,12]. Together, these mechanisms (i.e., elevated rates of evolution, rapid gene turnover, and variation in expression patterns) contribute to the observed differences in toxin composition among cone snails (as described above) and other venomous species (e.g., snakes [14,15,16]).

Cone snails are trophic specialists that show strong interspecific differences in diet [17,18,19,20,21,22,23,24]. Given that members of the Conidae differ in prey utilization patterns, interspecific differences in venom composition presumably reflect the evolution of venoms for use on different prey. However, while the mechanisms responsible for the observed differences in venom composition among species (as discussed above) have been elucidated, which of these mechanisms contribute to the differentiation of venoms among populations of widespread species that differ in prey utilization patterns remains unknown.

*Conus miliaris* is a widely distributed cone snail inhabiting tropical and subtropical waters from the eastern shores of Africa in the Indian Ocean to Easter Island in the southern Pacific [25]. Throughout its range, *C. miliaris* co-occurs with up to 36 congeners [26] and is a prey specialist that primarily feeds on just a few species of eunicid polychaetes [25]. However, at Easter Island, where congeners are largely absent, *C. miliaris* has undergone ecological release and feeds on a much more diverse assemblage of prey, including additional species of eunicids and members of nine other polychaete families [25]. The expanded dietary breadth of *C. miliaris* at Easter Island offers an excellent opportunity to evaluate the impacts of different feeding ecologies on the evolution and differentiation of venoms and to identify the molecular mechanisms contributing to this differentiation. 

Previous investigations of patterns of genetic differentiation of populations of *C. miliaris* in the Indo-West Pacific, based on analyses of sequences of regions of the mitochondrial cytochrome oxidase I gene and two O-superfamily conotoxin loci, illustrate that most populations exhibit low levels of genetic differentiation [27,28]. The inferred high levels of population connectivity are presumably achieved via high rates of gene flow accommodated by a planktonic larval phase that exists up to three weeks [29]. Nonetheless, the population at Easter Island is considerably genetically differentiated from other populations and levels of differentiation are greater for the conotoxin genes than for the mitochondrial locus, a pattern that was interpreted to reflect low levels of gene flow between populations at Easter Island and elsewhere in the Indo-West Pacific, and strong selection at conotoxin loci in response to dietary differences [27,28].

To determine patterns of genetic variation and patterns of variation in gene expression among populations of *C. miliaris*, we characterized venom duct transcriptomes of individuals of this species from three locations in the Indo-West Pacific (Guam, American Samoa, and Easter Island). After assembling and annotating transcripts, we categorized transcripts into two classes: those that show homology to genes encoding peptides that are known venom components (toxin-related) and all other transcripts (non-toxin-related) that presumably represent mRNAs of “house-keeping” genes. We evaluated these transcript sets to determine if populations exhibit more differentiation at transcripts encoding venom components than at other transcripts. In addition, while past work has shown that the population at Easter Island has a wider dietary breadth than populations elsewhere, we characterized diets of individuals from these three locations using a DNA-based approach to enable quantitative measures of dietary breadth and differences in prey utilization among populations and to determine if individuals at Easter Island are specialists for particular prey species. Moreover, we present diet data for populations at Guam and American Samoa as these were previously unavailable. We specifically sought to determine how dietary differences are associated with structural and regulatory differentiation at toxin-related and non-toxin-related transcripts among populations. Although we assume that many of the transcripts encoding venom components are utilized in venoms for capturing prey, several recent studies have shown that some cone snail species utilize their venom to deter predation and that offensive venoms differ considerably from defensive ones [30,31]. Moreover, the three locations are likely to differ in biotic and abiotic characteristics that may present distinct challenges for the populations of *C. miliaris* at these sites. Hence, patterns of variation observed for transcriptomes may be due to other factors and not necessarily result from differences in diet.

## 2. Results

### 2.1. Dietary Characterizations

We obtained 170 16S-rDNA sequences from feces or regurgitated worms from individuals of *C. miliaris* from Guam, American Samoa, and Easter Island. These included 59 sequences from feces of 58 specimens of *C. miliaris* at Guam (including two sequences recovered from feces of one individual), 21 sequences from 21 specimens at American Samoa, and 90 sequences from 68 specimens at Easter Island (including two sequences recovered from regurgitated worms, two obtained from two prey items present in the feces of one individual, 38 from feces recovered from two feeding bouts of 19 individuals, and three from three feeding bouts of one individual) (GenBank accession numbers MH634087-MH634256). In gene trees, recovered sequences occurred in 15 tip clades that were interpreted to represent 15 species of polychaetes (Table 1, Supplementary Figure S1). Sequences in all clades except one (Eunicidae ‘X8’) differed at only a few nucleotide sites. While only three and four prey species in the family Eunicidae were recovered from feces from individuals at Guam and American Samoa, respectively, sequences representing 10 species (nine species of eunicids (families Eunicidae, Onuphidae, and Lumbrineridae), one species of nereid and one capitellid species) were recovered from individuals at Easter Island. As indicated above, we examined fecal materials from separate feeding bouts for 20 individuals at Easter Island (two feeding bouts for 19 individuals and three feeding bouts for one individual). These prey items represented different species in all but five cases.

Only one prey item was shared among all populations (Eunicidae ‘X8’). Although most individuals at American Samoa utilized this taxon (~60%), it was less frequently recovered at Guam (~8% of prey items consumed) and Easter Island (~2%). All other inferred prey species, including the most commonly detected prey species at Guam (Eunicidae ‘X6’, ~67%) and Easter Island (*Palola* A1, ~38%), were unique to single locations. Estimates of dietary overlap (PS_I_ values) among populations ranged from 0.023 to 0.083 and were less than all values calculated from resampled data (lowest value obtained was 0.354) (i.e., *P* < 10^6^). The Shannon diversity index calculated for the Easter Island population (1.77) was nearly twice as large as those for populations from American Samoa (0.93) and Guam (0.98) (Table 1). In addition, average Kimura 2-parameter distances among sequences of prey from Easter Island (0.321) were about two- to three-fold greater than those from American Samoa (0.161) and Guam (0.114) (Table 1).

### 2.2. Sequence Datasets, de novo Transcriptome Assembly and Transcript Annotation

We generated 349,293,902 paired-end 200 bp reads from 22 *C. miliaris* individual cDNA libraries in a single flow cell lane averaging 15,876,996 (± 2,052,016) reads per individual (Table 2). After digital normalization, 22,293,902 paired-end reads were assembled using the Trinity de novo assembler resulting in a reference transcriptome for *C. miliaris* of 204,951 transcripts. Despite being a known issue with short-read assemblies [32], the larger number of transcripts in our *C. miliaris* assembly is surprising given the number of genes recently described from molluscs such as *Lottia giganeta* (23,851; Lotgi v1.0) and *Pinctada fucata* (23,257 [33]). However, many of the transcripts were either not annotated or identified as non-metazoan, likely representing bacteria, fungi or unicellular eukaryotes (i.e., possible contaminants). Additionally, as this assembly represented a combination of individuals from genetically distinct populations (see below), the high number of transcripts may represent allelic variation or different regions of the same genes that are not overlapping or combined during assembly [32].

For Easter Island, Guam, and American Samoa population transcriptomes, 131,525,916, 130,566,380, and 87,201,606 paired-end reads were assembled into 149,583, 134,741, and 98,828 transcripts, receptively (Table 2). Given the smaller number of individuals included in the American Samoa population assembly (i.e., six as compared with eight in the Easter Island and Guam assemblies), the lower number of transcripts produced by this assembly is not surprising. However, when assemblies were conducted on an individual basis, the number of transcripts recovered for each of the 22 individuals was similar across populations (data not shown). Statistics (e.g., number of transcripts, average length of transcripts, N50, etc.) for each assembly are presented in Table 2. 

**Figure 1:**
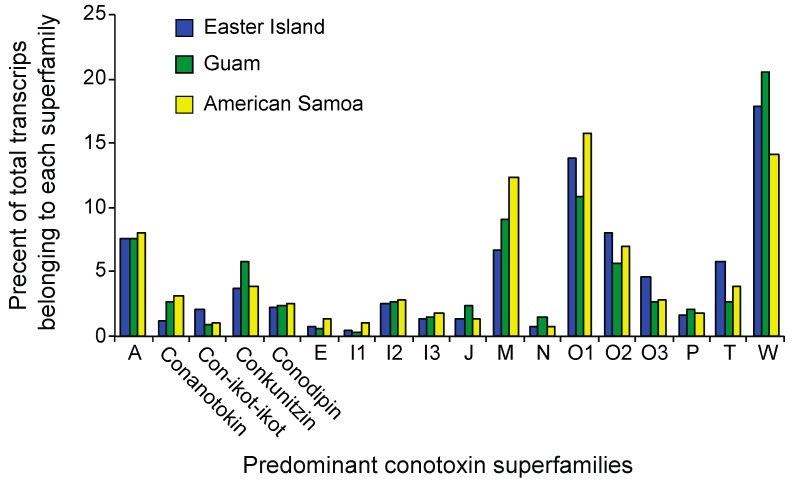
The percentage of toxin-related transcripts recovered belonging to each of the major conotoxin superfamilies from populations of *C. miliaris* from Easter Island, Guam, and American Samoa. These transcripts make up 85, 81, and 86 percent of toxin-related transcripts recovered from Easter Island, Guam, and American Samoa, respectively.

The *C. miliaris* reference transcriptome as well as population transcriptomes were annotated using BLASTx searches against locally curated databases consisting of conotoxins, annotated proteins from *Aplysia californica* and *Lottia gigantea* genomes, known *Conus* proteins, as well as the UniProt protein database. Overall, 31,099 (i.e., ~15%) of the transcripts from the *C. miliaris* reference transcriptome had similarity (< E-values 1 × 10^−5^) with known proteins or conotoxins in the reference databases (Table 2). Of these 31,099 annotated transcripts, 516 were identified as being toxin-related. For the population level assemblies, an average of 21,808 transcripts had similarity to proteins in our BLAST databases with 435, 340, and 284 transcripts being identified as being toxin-related in the Easter Island, Guam, and American Samoa assemblies, respectively (Table 2). When toxin-related transcripts are categorized into superfamilies, no obvious population level differences are seen (Figure 1). We evaluate whether or not populations exhibit differences in toxin transcript composition below the level of superfamilies through examination of patterns of expression as discussed below.

### 2.3. Differential Expression

**Figure 2:**
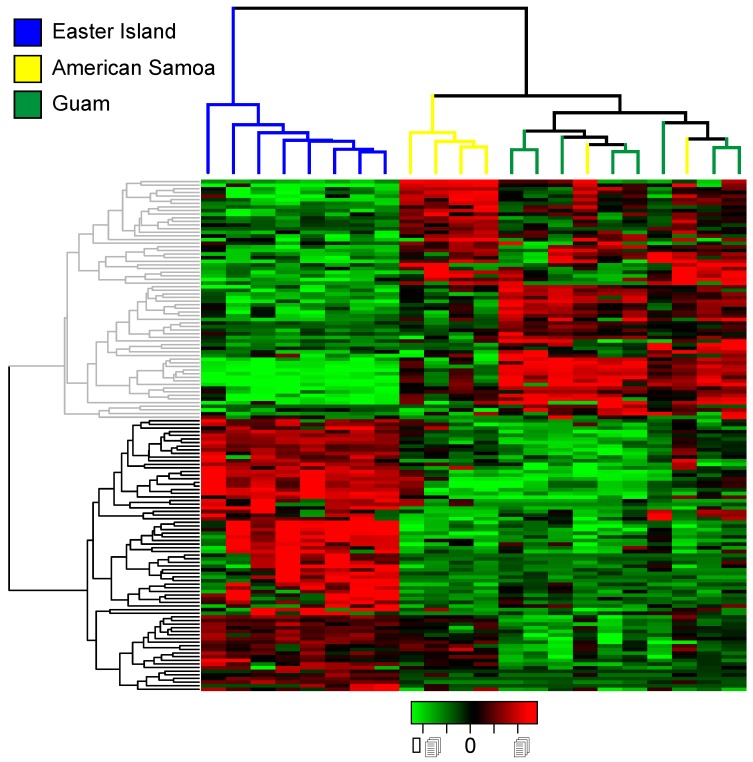
Heat map showing differences in toxin-related transcript expression between individuals of *C. miliaris* from Easter Island, Guam, and American Samoa based on replicate samples. Statistically significant differentially expressed transcripts were identified using edgeR at *P* = 0.001, with a minimum four-fold difference in expression. Expression values (FPKM) are log_2_-transformed and median centered so that green represents under-expressed transcripts and red represents over-expressed transcripts. Unsupervised hierarchical clustering of the 22 *C. miliaris* individuals according to their transcript expression patterns is shown above the heat map. Similarly, unsupervised hierarchical clustering of transcripts with similar expression patterns is presented to left of heat map (Supplementary Tables S1 and S2).

On average, approximately three million quality trimmed, single-end reads from each individual were successfully mapped to the annotated *C. miliaris* reference transcriptome. Of the 31,099 annotated *C. miliaris* transcripts, 4694 were found to be differentially expressed among individuals. Of the 30,543 annotated transcripts not related to toxins, 4187 were found to be differentially expressed. In both cases, hierarchical clustering failed to differentiate between populations. However, when only toxin-related transcripts were analyzed, 141 transcripts were differentially expressed among individuals. Furthermore, based on hierarchical clustering of the differentially expressed toxin-related transcripts, expression patterns of individuals at Easter Island were differentiated from those of individuals at Guam and American Samoa (Figure 2, Supplementary Tables S1 and S2). When we only considered individuals from Guam and American Samoa, eight transcripts were differentially expressed among individuals and hierarchical clustering did not separate individuals from the two locations (results not shown). We calculated the coefficients of variation (CV) of expression values of toxin-related transcripts of the three populations to determine if individuals from the Easter Island population show greater variation in expression levels than individuals from populations elsewhere. On the contrary, levels of variation were similar among the three populations; slightly more transcripts at Guam (157) showed higher CV values than at either Easter Island (155) or American Samoa (133).

### 2.4. Single Nucleotide Polymorphisms, Diversity, and Population Structure

The initial SNP screening identified 585,736 high-quality SNPs. Of these, 26,920 (i.e., 4.6%) had confident genotypes for all 22 individuals. The multiplexing of 22 individuals in a single HiSeq lane likely accounts for the low percentage of confidently genotyped SNPs; the most highly expressed genes (e.g., conotoxins) were likely sequenced to a sufficient depth to be confidently genotyped for all individuals. Examination of the levels of diversity exhibited at these SNPs revealed that while non-toxin-related SNPs show similar levels of diversity among the three populations, the population at Easter Island shows greater transcript diversity at toxin-related SNPs than do the other populations (Table 3).

**Figure 3:**
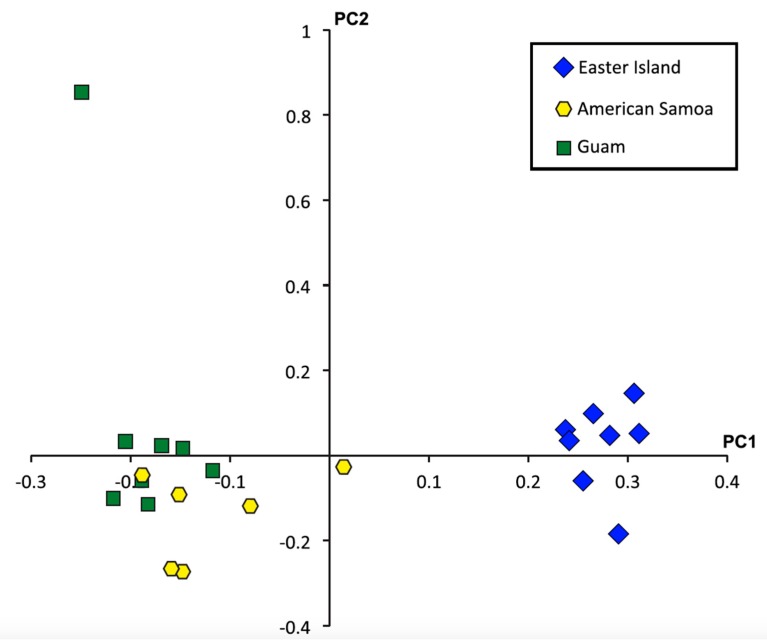
Principle components plot based on analyses of 26,920 single nucleotide polymorphisms (SNPs) with high quality genotypes for all 22 individuals. PC axis 1 is significant (*P* = 2.48 × 10^−15^).

A principal component analysis of the 26,920 SNPs genotyped from all individuals suggests strong genetic differentiation between the Easter Island population and the Guam and American Samoa populations (Figure 3). Pairwise F_ST_ values for all 26,920 SNPs were lower than, but comparable to those reported for the mitochondrial cytochrome oxidase I gene [27]. Values between Easter Island and Guam as well as Easter Island and American Samoa indicted higher genetic differentiation than between Guam and American Samoa (Table 4). Furthermore, while F_ST_ values based on the 24,557 non-toxin-related SNPs indicated little genetic differentiation between populations, the 2363 toxin-related SNPs exhibited strong genetic differentiation, especially between the Easter Island population and the American Samoa and Guam populations (Table 5). Nonetheless, all pairwise comparisons revealed four-fold to five-fold greater differences in F_ST_ values calculated for toxin-related SNPs than for non-toxin-related SNPs.

### 2.5. Data Accessibility

Raw NGS sequence data for each individual are available from NCBI SRA under accession numbers PRJNA257931 and SRP045405. Assembled transcripts are available from Dryad for *C. miliaris* as a whole, as well as for each geographic population, as FASTA files under accession number: 10.5061/dryad.t74q4. Relative expression levels are available from Dryad as tab-delimited files corresponding to the number of reads mapping back to each annotated transcript of the *C. miliaris* assembly and FPKM values for each of the 22 individuals, geographic populations, as well as for *C. miliaris* under accession doi: 10.5061/dryad.t74q4. Putative single nucleotide polymorphisms (SNPs) are available from Dryad as a VCF file under accession number: 10.5061/dryad.t74q4.

## 3. Discussion

Populations of *C. miliaris* exhibit substantial differences in diets in terms of the particular species preyed upon and dietary breadth at different locations (Table 1, Supplementary Figure S1). However, while resource use at Easter Island has expanded, individuals at Easter Island do not appear to show specializations for particular prey taxa. Although the wider dietary breadth at Easter Island is not associated with an increased diversity of venom components expressed (Table 2, Figure 1), this population exhibits a higher level of genetic variation at toxin-related genes than those from Guam and American Samoa (Table 3). Additionally, venom composition, in terms of gene expression patterns and levels of structural divergence, is most strongly differentiated at Easter Island (Table 5, Figure 2). Finally, while populations at Guam and American Samoa exhibit similar dietary breadths but otherwise largely prey on different polychaete species, toxin-related transcripts show a greater degree of differentiation than do other annotated transcripts (Table 3 and Table 5). However, these populations do not exhibit differences in expression patterns of toxin-related transcripts (Figure 2). The significance and implications of these results are discussed below.

### 3.1. Diets and Dietary Breadth

Populations of *C. miliaris* at Guam, American Samoa, and Easter Island show little overlap in prey utilization and, as anticipated from Kohn’s earlier work on the diets of this species [25], the population at Easter Island has a wider dietary breadth than populations at other locations where members of the polychaete family Eunicidae are the predominant prey type (Table 1, Supplementary Figure S1). Because we identified more prey items from individuals at Easter Island (N = 90) than at Guam (N = 59) or American Samoa (N = 21), differences in the number of prey species identified may reflect the sample sizes used. Nonetheless, although the sample size for the population at Guam is nearly three times larger than that at American Samoa, dietary diversity statistics are comparable for these populations. 

Populations with wide niche breadths, such as the population of *C. miliaris* at Easter Island, typically consist of (i) generalist individuals with similarly broad niche breadths (i.e., population and individual niche widths are similar), (ii) sets of specialist individuals that each utilize a subset of available resources (i.e., niche widths of individuals are non-overlapping and narrower than the population’s niche width), or (iii) sets of generalist and specialist individuals that differ in niche width (i.e., niche widths of some individuals are nested within the niche widths of others) [34,35,36,37]. As multiple taxa were identified from fecal materials recovered for separate feeding bouts of several *C. miliaris* individuals (Table 1, Supplementary Figure S1), individuals at Easter Island do not appear to exhibit specializations for particular prey. Unfortunately, an insufficient number of observations was obtained to determine the level of dietary specializations of individuals. Because species diversity and population densities of polychaetes at Easter Island are comparable to those at other locations in the Indo-West Pacific [38], differences in dietary breadth are not likely to be due to differences in the density or diversity of available prey at the locations examined, but instead may be due to ecological release at Easter Island [25].

The feeding ecologies of the three populations examined are all distinct with very little overlap in resource use (Table 1, Supplementary Figure S1). While individuals at Easter Island prey on eunicid, onuphid, lumbrinerid, nereidid, and capitellid polychaetes, the diets of individuals at Guam and American Samoa consist solely of eunicids (Table 1, Supplementary Figure S1). Given this, it is not surprising that measures of dietary breadth at Guam and American Samoa are less than those for the population at Easter Island. These patterns of variation provide a framework to understand how differences in feeding specialization (as exhibited by all populations) and differences in dietary breadth (as exhibited by the population at Easter Island in comparison with populations at Guam and American Samoa) are associated with regulatory and structural changes among venom genes utilized to capture prey.

### 3.2. Mechanisms Contributing to the Differentiation of Venoms among Populations with Different Resource Use Patterns

Previous analyses of patterns of variation exhibited at two polymorphic conotoxin loci revealed significant differences in allelic frequencies at Easter Island, while populations at Guam and American Samoa showed no evidence of differentiation [28]. In addition, a phylogeographic study of *C. miliaris* based on an analysis of mitochondrial cytochrome oxidase I sequences suggested that the population at Easter Island is genetically isolated from populations elsewhere in the Indo-West Pacific and that other populations in this region were not genetically structured [27]. A much more expanded survey of genetic variation exhibited by the entire venom duct transcriptomes reveals similar trends (Table 4 and Table 5 and Figure 3). Indeed, the population at Easter Island is the one that is most strongly differentiated at both toxin and non-toxin-related transcripts from other populations. Nonetheless, while the previous study of the two conotoxin loci showed no differentiation among populations at Guam and American Samoa, our results reveal that toxin-related transcripts show greater levels of divergence than non-toxin-related transcripts at these populations (Table 5). Given this, the differences in diet observed for these populations may drive divergence at toxin encoding genes through selection for effective capture of different prey types, although other explanations could be feasible. For instance, although cone snails utilize venoms to subdue and capture prey, venoms also serve a defensive role in some species [30,31]. Although we do not know if *C. miliaris* utilizes its venom defensively nor how predation pressures on this species differ among locations, it is possible that these or other factors are responsible for the patterns of variation that we observed. Future studies aimed at understanding patterns of variation in predation pressures and identifying defensive and offensive components of venoms of *C. miliaris* would help to address this issue.

Populations with expanded niche breadths are proposed to exhibit greater phenotypic variation than populations with narrow niche breadths when individuals exhibit specializations for subsets of resources [34,36,39,40,41]. Here, we can specifically compare intraspecific patterns of genetic variation of genes associated with resource use for populations with different niche breadths. Across phylogenetically disparate cone snail species, there is a positive relationship between venom composition complexity and dietary breadth [42]. Within species however, wider dietary breadth does not appear to be associated with any large shifts in the major types of venom components expressed (Figure 1). Nonetheless, our family-level assignment of toxin types may obscure shifts in expression within these families. Indeed, individuals at Easter Island share more similar patterns of expression of toxin-related genes than do individuals from Guam and American Samoa based on estimates of coefficients of variation of toxin-related transcript expression values (see also Figure 2). On the other hand, the population at Easter Island exhibits greater transcript diversity at toxin-related transcripts than populations at Guam and American Samoa, while measures of diversity at non-toxin-related transcripts are comparable among the three populations (Table 3). These results align with a number of studies revealing a strong correlation between dietary breadth and conotoxin gene diversity [42,43,44,45]. Although we cannot attribute these patterns to the evolution of individual-level feeding specializations at Easter Island, they at least demonstrate that changes in feeding breadth are associated with structural changes at toxin-related genes.

The population of *C. miliaris* at Easter Island is distinct from other populations in terms of structural and regulatory divergence of toxin-related transcripts as well as its wider dietary breadth and lack of genetic connectivity with other populations in the Indo-West Pacific. Populations at Guam and American Samoa differ in diet, but have similar dietary breadths, show similar toxin-related transcript expression patterns, and apparently experience high levels of gene flow with each other. Although populations at Guam and American Samoa show greater differentiation at toxin-related than at non-toxin-related transcripts, the level of differentiation is much less than found for comparisons involving Easter Island. Differences in diet are associated with the genetic differentiation of conotoxin loci among the three population comparisons. Nonetheless, the extent of this differentiation is greatest when populations show large differences in dietary breadth and low levels of genetic connectivity. Because populations at Guam and American Samoa show similar conotoxin gene expression patterns despite large differences in diet, differences in conotoxin expression patterns appear to only be associated with differences in dietary breadth and lack of genetic connectivity of populations (i.e., at Easter Island), but not differences in diet. A similar association between the extent of differentiation of conotoxin genes and dietary breadth was also observed for *C. ebraeus*, another worm-eating species that exhibits geographical variation in feeding ecology [44]. Given these observations (i.e., differences in diet are associated with differentiation at toxin-related transcripts but not always associated with differences in expression patterns), changes in allelic frequencies and not changes in gene expression patterns may be responsible for divergence in venom composition among populations of cone snails that show differences in diet. Large changes in gene expression patterns may occur with shifts in dietary breadth, especially for shifts that involve an increase in the phylogenetic disparity of prey, or when populations are genetically isolated (i.e., gene flow with other populations is low). Given that we cannot disentangle how diets, dietary breadth, and gene flow are associated with mechanisms that facilitate or limit opportunities for the differentiation of venoms, future studies should explore patterns of variation of venoms among populations with different dietary breadths and high levels of gene flow, and with similar dietary breadths and low levels of gene flow.

## 4. Conclusions

To elucidate the contributions of predator-prey interactions to intraspecific genetic and phenotypic divergence, we characterized diets and applied a comparative transcriptomic approach to three populations of the cone snail *C. miliaris.* While the three populations showed very little overlap in prey utilization, the population at Easter Island exhibited a much broader dietary breadth than populations at American Samoa and Guam. Contrary to previous studies, no associations between particular conotoxin gene superfamilies or total conotoxin diversity and prey type were observed, supporting the hypothesis that differences in prey do not correlate to changes in gene superfamily size or total conotoxin diversity [43,44]. However, differentiation of conotoxin genes and changes in their expression patterns appear to be associated with differences in prey utilization and dietary breadths of populations. Such differentiation may be indicative of directional selection as a consequence of ecological pressures (i.e., dietary breadth) [46,47], a phenomenon that has been suggested to be important in early stages of divergence among populations [48,49].

## 5. Materials and Methods

### 5.1. Dietary Analyses

Specimens of *C. miliaris* were collected at locations in Guam, American Samoa, and Easter Island and placed in small containers with seawater within one to two hours after collection. Fecal or regurgitated materials were recovered and preserved in 95% ethanol. A mark-recapture study was performed at Easter Island, allowing for the recovery of feces from multiple feeding bouts from a subset of individuals. Feces were examined via microscopy to guide the DNA-based prey determinations. In particular, presence of multiple pairs of jaws, jaw parts, and either types of setae or acicula were used to infer the number of prey taxa present in feces. Worm fragments or small sections of regurgitated worms were removed from feces (one fragment from feces that appeared to contain a single prey item and multiple fragments (up to five) for feces that appeared to contain more than one prey item) and used for genetic identification. 

Total genomic DNA was extracted from collected material using the EZNA Mollusc DNA extraction kit (Omega Bio-tek, Norcross, USA) following the manufacturer’s recommendations. A combination of previously reported annelid specific primers (forward primers 16SANNf2 and 16SANNf3, reverse primer 16Spr1 [50]), a newly designed annelid specific forward primer (16SANNf4: GATATTTTAACCGTGCTAAGGTAGCG) and universal 16S primers (16Sar and 16Sbr [51]) were utilized to amplify an ~300–350 bp region of the mitochondrial large ribosomal subunit (16S-rDNA) gene. Reactions were conducted in 10 ul reactions under the following conditions: 40 cycles of 94 °C for 30 s, 60 °C for 30 s and 72 °C for 30 s. 

Successfully amplified products were diluted 1:5 in deionized water and submitted for sequencing at the University of Michigan DNA Sequencing Core facilities. Ambiguities in chromatograms were corrected by comparison with the complementary DNA strand in Sequencher v5.0 (Gene Codes Corporation, Ann Arbor, USA). Sequences were manually aligned using Se-Al v2.0a11 [52] with several hundred annelid sequences obtained from GenBank. To delimit polychaete prey species, neighbor-joining trees were constructed under an appropriate model of evolution and 500 bootstrap replicates using MEGA v5.2.1 [53]. Given the difficulties of aligning 16S-rDNA sequences across major polychaete taxonomic categories, subsets of data for each major taxon were analyzed separately with tip clades of sequences inferred to represent distinct polychaete species. 

Levels of dietary overlap among populations were evaluated by estimating proportional similarity indices (PS_I_ values [54]) from species utilization frequencies. Statistical significance of similarity indices was evaluated using a Monte Carlo simulation approach with one million resampled datasets. Levels of prey diversity of each population were measured using Shannon diversity indices [55] and average genetic distances among sequences of prey as a proxy for the phylogenetic disparity of prey. We evaluated levels of resource specialization of individuals at Easter Island based on comparisons of prey items taken during separate feeding bouts.

### 5.2. De novo Transcriptome Assembly and Annotation

Detailed descriptions of sample collection, library preparation and sequencing, as well as transcriptome assembly and annotation are described previously in Genomic Resources Development Consortium [34]. Briefly, eight, eight, and six individuals of *C. miliaris* were collected from Easter Island, Guam, and American Samoa, respectively. Total RNA was extracted from 22 dissected venom ducts and prepared for sequencing in a single flowcell lane on an Illumina HiSeq 2000 at the University of Michigan DNA Sequencing Core.

Quality scores, nucleotide distributions, and the overall quality of the 100 bp paired-end reads were examined using the FASTX Toolkit [56]. Given the high quality of the data and the fact that over-aggressive quality trimming can negatively affect the assembly of RNA-seq data [57] (D.A. Weese personal observation), the reads were not filtered or quality-trimmed prior to assembly. For the *C. miliaris* reference transcriptome, reads from all 22 individuals were combined and digitally normalized [58] prior to assembly using Trinity (version Trinityrnaseq_r20140413p1) [59]. For the individual location assemblies, raw reads were pooled based on sampling location (i.e., Easter Island, Guam or American Samoa) and assembled using Trinity under default parameters. 

To annotate the de novo assemblies for each dataset (whole species and three geographic populations), transcripts were first searched against a locally curated databases of non-redundant conotoxin and conotoxin signal sequences downloaded from the Conoserver database [60] using BLASTx (version 2.2.29) with default parameters and retaining matches with an *e*-value < 10^−5^. Transcripts that failed to match any toxins in our conotoxin database were then compared to local databases comprised of: (1) annotated proteins from the two mollusc species available on the NCBI Unigene database, *Aplysia californica,* and *Lottia gigantea*; (2) all *Conus* related proteins downloaded from NCBI’s nr database; and (3) the UniProt protein database using BLASTx with an *e*-value cut off of 1 × 10^−5^. The assemblies were then divided into two sets: transcripts that were toxin-related, which are those transcripts that had homology with proteins in our conotoxin database and those that were annotated but had no homology to proteins in our conotoxin database (i.e., not toxin-related). Toxin-related transcripts were assigned to superfamilies based on their best hits in the BLASTx search against the conotoxin database.

### 5.3. Differential Expression

Annotated transcripts from the reference *C. miliaris* assembly were used as the reference transcriptome for the evaluation of expression values and SNP detection. Prior to mapping, reads were quality trimmed based on a phred-scale quality score cut-off of 20 with reads shorter than 20 bases being discarded using the FASTX Toolkit. Trimmed, single-end reads for each individual were then individually mapped against the reference transcriptome with Bowtie v1.0.1 [61] using the parameters --all --best --strata -m 300 --chunkmbs 512. On the basis of these alignments, RSEM v1.2.9 [62] was used with default parameters to estimate the expression level of annotated transcripts. Differential expression analyses were conducted on three subsets of the data: (1) all *C. miliaris* annotated transcripts, (2) *C. miliaris* transcripts not related to toxins, and (3) toxin-related *C. miliaris* transcripts following the methods outlined by Hass et al. [63]. For each population, individuals were treated as biological replicates and expression values were normalized by the trimmed mean of M values (TMM) method using edgeR [64]. The differential expression values were calculated using the empirical Bayes estimation approach using the edgeR package [65] in Bioconductor (http://bioconductor.org). For visualization, transcripts that showed more than fourfold differences in expression with a false discovery-corrected statistical significance of <0.001 were hierarchically clustered using the heatmaps2 package in R. To evaluate patterns of variation in expression of toxin-related transcripts of the three populations, we calculated the coefficients of variation of expression values and determined if more loci show higher coefficients of variation at Easter Island than do the other populations. 

### 5.4. Single-Nucleotide Polymorphism (SNP) Identification, Diversity, Population Structure, and Outlier Analysis

For SNP detection, trimmed paired-end reads for each individual were mapped to the *C. miliaris* reference transcriptome using Bowtie2 v2.1.0 [66] with the following parameters: --no-unal --local -p 6. After the duplicate reads in the alignments were marked with the MarkDuplicates utility of Picard [67], SNPs were identified using the Genome Analysis Toolkit [68,69] following the recommendations of the Broad Institute’s best practices [70].

A principal component analysis (PCA) was conducted on high quality SNPs (Q > 20) using the EignSoft package [71] to investigate the population structure between individuals. The SNP dataset was further trimmed to exclude any variant sites that were genotyped heterozygous for all individuals (likely due to paralogous genes or contamination [32]) and any site in which the minor allele frequency was less than 2%. Measures of transcript diversity (Qinter) and pairwise F_ST_ values for all SNPs, non-toxin-related SNPs, and toxin-related SNPs were calculated in GenePop [72].

## Figures and Tables

**Table 1:** Dietary data for individuals of *C. miliaris* from locations in the Indo-West Pacific (see Supplementary Figure S1).

Prey Item/Statistic	Easter Island	Guam	American Samoa
Eunicidae (total)	76	59	21
Eunicidae ‘X6’		40	
Eunicidae ‘X7’		10	
Eunicidae ‘X8’	2	4	13
Eunicidae ‘X9’	13		
Eunicidae ‘X10’	5		
Eunicidae ‘X13’	19		
Eunicidae ‘X15’	2		
Eunicidae ‘X16’			4
Eunicidae ‘X18’			4
*Palola* A1	35		
*Palola* A3		5	
Onuphidae (‘O1’)	4		
Lumbrinereidae (‘L1’)	1		
Nereididae (‘N2’)	8		
Capitellidae (‘C1’)	1		
Number of prey items identified	90	59	21
Inferred number of species	10	4	3
Inferred number of families	5	1	1
Shannon diversity index of prey	1.77	0.98	0.93
Average genetic distances among prey	0.321	0.114	0.161

**Table 2:** Summary for the assembly and annotation of the *C. miliaris*, Easter Island, Guam, and American Samoa transcriptomes.

Statistic	*C. miliaris*	Easter Island	Guam	American Samoa
Number of PE reads	349,293,902	131,525,916	130,566,380	87,201,606
Total transcripts	204,951	149,583	134,741	98,828
Average transcript length (bp)	624.2	560.4	550.5	428.9
Longest transcript (bp)	25,902	23,251	16,001	15,460
N50 (bp)	879	719	693	463
Annotated transcripts (%)	31,099 (15.2)	23,166 (15.5)	24,303 (16.3)	17,956 (18.2)
Toxin-related transcripts	516	435	340	284
% of total reads mapped to toxin-related transcripts	66	59	72	65

**Table 3:** Estimates of transcript diversity of populations of *C. miliaris* based on analyses of all 26,920 SNPs, 24,557 non-toxin related SNPs and 2363 toxin-related SNPs.

Population	All SNPs	Non-Toxin-Related SNPs	Toxin-Related SNPs
Easter Island	0.159	0.155	0.198
Guam	0.153	0.153	0.149
American Samoa	0.157	0.157	0.155

**Table 4:** Pairwise F_ST_ values as measures of genetic differentiation between populations of *C. miliaris* based on analyses of mitochondrial cytochrome oxidase I sequences (above diagonal; from [27]) and all SNPs (below diagonal).

Population	Easter Island	Guam	American Samoa
Easter Island	-	0.121	0.143
Guam	0.069	-	−0.012
American Samoa	0.054	0.001	-

**Table 5:** Pairwise F_ST_ values as measures of genetic differentiation between populations of *C. miliaris* based on 24,557 non-toxin-related SNPs (above diagonal) and 2363 toxin-related SNPs (below diagonal).

Population	Easter Island	Guam	American Samoa
Easter Island	-	0.050	0.034
Guam	0.207	-	0.008
American Samoa	0.178	0.035	-

## Supplementary Materials

The following are available online at https://www.mdpi.com/2072-6651/11/5/299/s1. Figure S1: Neighbor-joining trees constructed with Kimura 2-parameter distances (Kimura 1980) from alignments of sequences recovered from feces or regurgitated worms (bold typeface) from Guam (G), American Samoa (AS), and Easter Island (EI) and polychaete sequences from GenBank (regular typeface; accession numbers included in names), Table S1: Differentially expressed toxin-related transcripts showing similar expression patterns of up-regulation in individuals of *C. miliaris* from Guam and American Samoa and down-regulation for individual from Easter Island, Table S2: Differentially expressed toxin-related transcripts showing similar expression patterns of up-regulation in individuals of *C. miliaris* from Easter Island and down-regulation from individual from Guam and American Samoa.
