# Supplementary material for: Effects of Predator-Prey Interactions on Predator Traits: Differentiation of Diets and Venoms of a Marine Snail
Source: Toxins (Basel). 2019 May 25;11(5):299. doi: 10.3390/toxins11050299 (PMC6563511; doi:10.3390/toxins11050299)
Supplement: Supplementary file 1 [file toxins-11-00299-s001.pdf]

Effects of Predator-Prey Interactions on Predator Traits: Differentiation of Diets and Venoms of a Marine Snail

David A. Weese and Thomas F. Duda, Jr.

SUPPLEMENTARY INFORMATION:

Table S1. Differentially expressed toxin-related transcripts showing similar expression patterns of up-regulation in individuals of *C. miliaris* from Guam and American Samoa and down regulation from individual from Easter Island.

Transcript	Annotation	Conoserver ID
c35391_g1_i1	Mr22.1 precursor *Conus marmoreus*	P05506
c43925_g2_i1	Unidentified protein *Conus caracteristicus*	P05240
c67316_g1_i1	Cl14.8 precursor *Conus californicus*	P04024
c69481_g1_i1	G21.1 precursor *Conus geographus,* Geography cone	P05803
c69481_g1_i3	G21.1 precursor *Conus geographus,* Geography cone	P05803
c72241_g1_i3	Eu14.7 precursor *Conus eburneus*	P04504
c74125_g1_i2	Cl6.13 precursor *Conus californicus*	P04029
c74627_g2_i3	conkunitzin Pu1 precursor *Conus pulicarius*	P05693
c76925_g2_i2	Unidentified protein *Conus caracteristicus*	P05240
c78783_g2_i1	Mr105 precursor *Conus marmoreus*	P05508
c79440_g1_i1	con ikot ikot precursor *Conus striatus,* Striated cone	P03890
c79440_g2_i1	G21.1 precursor *Conus geographus,* Geography cone	P05803
c79440_g2_i2	con ikot ikot G5 precursor *Conus geographus,* Geography cone	P05801
c79440_g2_i4	G21.1 precursor *Conus geographus,* Geography cone	P05803
c79440_g2_i5	G21.1 precursor *Conus geographus,* Geography cone	P05803
c81265_g1_i1	Cl6.13 precursor *Conus californicus*	P04029
c81265_g2_i6	Cl6.13 precursor *Conus californicus*	P04029
c81966_g1_i1	TxIXA precursor *Conus textile* Cloth of gold cone	P00608
c81966_g1_i2	Pu9.3 precursor *Conus pulicarius*	P05642
c81966_g1_i3	Pu9.3 precursor *Conus pulicarius*	P05642
c81966_g1_i6	TxIXA precursor *Conus textile,* Cloth of gold cone	P00608
c83925_g1_i3	Tx6.5 *Conus textile,* Cloth of gold cone	P05835
c83925_g1_i5	Tx6.5 *Conus textile,* Cloth of gold cone	P05835
c86092_g2_i7	conantokin Pu1 precursor *Conus pulicarius*	P05674
c87440_g1_i1	con ikot ikot G5 precursor *Conus geographus,* Geography cone	P05801
c76312_g3_i2	H Vc1 prepropeptide *Conus victoriae*	P06385
c78833_g1_i1	T superfamily conotoxin Vx5.2 precursor *Conus vexillum*	P06112
c1284_g2_i1	O1 M19 precursor *Conus miliaris*	P00604
c66936_g1_i1	M Vr3 TYN01 precursor *Conus varius*	P04865
c70689_g1_i1	O1 Ar6.19 precursor *Conus arenatus*	P00748
c75079_g2_i1	O1 Lt6e precursor *Conus litteratus*	P01158
c77403_g2_i1	O1 LiCr95 precursor *Conus lividus*	P01121
c77403_g1_i2	O1 Ar6.9 precursor *Conus arenatus*	P00738

c77403_g2_i2	O1 LiCr95 precursor *Conus lividus*	P01121
c78702_g3_i1	M Rt3 TP02 precursor *Conus rattus*	P04829
c80317_g2_i1	O1 Ar6.18 precursor *Conus arenatus*	P00747
c80565_g2_i1	O2 Contryphan Bt1 precursor *Conus betulinus*	P05916
c81778_g3_i2	A Pu1.1 precursor *Conus pulicarius*	P01180
c85006_g4_i1	A Im1.95 precursor *Conus imperialis*	P04066
c85006_g4_i2	A Im1.95 precursor *Conus imperialis*	P04066
c85067_g1_i6	I3 Ca11.3 precursor *Conus caracteristicus*	P03797
c87453_g2_i1	I2 Eu12.4 precursor *Conus eburneus*	P03861
c760_g1_i1	O1 Ar6.3 precursor *Conus arenatus*	P00726
c17083_g1_i1	M Eb2C04 precursor *Conus ebraeus*	P05115
c52129_g1_i3	M Mr038 precursor Conus marmoreus	P05510
c55015_g1_i1	O3 Pu6.37 precursor *Conus pulicarius*	P05636
c55015_g1_i2	O3 Pu6.37 precursor *Conus pulicarius*	P05636
c68235_g1_i1	O2 LtXVA precursor *Conus litteratus*	P01162
c68235_g1_i2	O2 Ca15a precursor *Conus caracteristicus*	P03864
c72308_g4_i1	O1 Om6.2 precursor *Conus omaria*	P03041
c72308_g4_i3	O1 Au6.1 precursor *Conus aulicus*	P03047
c72868_g1_i1	T Lt5i precursor *Conus litteratus*	P01144
c72868_g1_i2	T Lt5i precursor *Conus litteratus*	P01144
c73929_g1_i8	O1 Ca6.1 precursor *Conus caracteristicus*	P03129
c79247_g1_i2	D Cp20.4 precursor *Conus capitaneus*	P03592
c80036_g2_i4	I2 Eu11.3 precursor *Conus eburneus*	P03812
c80036_g2_i5	I2 Eu11.3 precursor *Conus eburneus*	P03812
c80565_g3_i1	O2 Contryphan Sm precursor *Conus stercusmuscarum*	P01311
c80565_g3_i2	M Eb2C11 precursor *Conus ebraeus*	P04813
c80565_g2_i3	O2 Di15.1 precursor *Conus distans*	P05937
c81778_g1_i1	A SrIA/SrIB precursor *Conus spurius*	P03751
c81778_g1_i2	A Bt1.5 precursor *Conus betulinus*	P02914
c81778_g1_i3	A Bt1.5 precursor *Conus betulinus*	P02914
c82440_g1_i2	J Mr14.1 precursor *Conus marmoreus*	P05887
c84431_g1_i1	O2 Vc6.14 precursor *Conus victoriae*	P04275
c87446_g3_i1	T Lt5h precursor *Conus litteratus*	P01146

Table S2. Differentially expressed toxin-related transcripts showing similar expression patterns of up-regulation in individuals of *C. miliaris* from Easter Island and down regulation from individual from Guam and American Samoa.

Transcript	Annotation	Conoserver ID
c28638_g1_i1	Unidentified protein *Conus caracteristicus*	P05240
c34086_g1_i1	Unidentified protein *Conus caracteristicus*	P05240
c43925_g1_i1	Unidentified protein *Conus caracteristicus*	P05240
c56073_g1_i1	G14.5 precursor *Conus geographus,* Geography cone	P05807
c62874_g2_i2	conophysin G precursor *Conus geographus,* Geography cone	P05809
c62874_g2_i3	conophysin G precursor *Conus geographus,* Geography cone	P05809
c66493_g1_i1	Mr15.1 precursor *Conus marmoreus*	P05486
c66493_g1_i2	Mr15.1 precursor *Conus marmoreus*	P05486
c66791_g1_i1	conkunitzin Pu2 *Conus pulicarius*	P05696
c67963_g1_i1	QcVIA *Conus quercinus*	P01512
c72241_g1_i4	Eu14.7 precursor *Conus eburneus*	P04504
c72357_g1_i1	Unidentified protein *Conus caracteristicus*	P05240
c72357_g1_i4	Unidentified protein *Conus caracteristicus*	P05240
c74542_g1_i1	G21.1 precursor *Conus geographus,* Geography cone	P05803
c75243_g1_i1	Conantokin Eu2 precursor *Conus eburneus*	P04568
c76571_g5_i4	Lt9a precursor *Conus litteratus*	P01172
c76925_g3_i3	Unidentified protein *Conus caracteristicus*	P05240
c76925_g3_i4	Unidentified protein *Conus caracteristicus*	P05240
c78837_g4_i1	G21.1 precursor *Conus geographus,* Geography cone	P05803
c79781_g1_i1	conkunitzin G1 precursor *Conus geographus,* Geography cone	P05805
c79781_g1_i2	conkunitzin G1 precursor *Conus geographus,* Geography cone	P05805
c79781_g1_i3	conkunitzin G1 precursor *Conus geographus,* Geography cone	P05805
c80066_g1_i3	Cal9.2c precursor *Conus californicus*	P04046
c80445_g4_i3	conkunitzin Pu2 precursor *Conus pulicarius*	P05695
c80937_g2_i2	contulakin Lt2 precursor *Conus litteratus*	P01176
c80937_g2_i4	contulakin G precursor *Conus geographus,* Geography cone	P01323
c80937_g2_i5	contulakin Lt2 precursor *Conus litteratus*	P01176
c81435_g1_i1	CaXVIIA precursor *Conus caracteristicus*	P02839
c83925_g4_i1	Tx6.5 *Conus textile,* Cloth of gold cone	P05835
c84096_g1_i1	Unidentified protein *Conus caracteristicus*	P05240
c84096_g2_i1	Unidentified protein *Conus caracteristicus*	P05240
c84096_g2_i6	Unidentified protein *Conus caracteristicus*	P05240
c84096_g2_i8	Unidentified protein *Conus caracteristicus*	P05240
c85542_g5_i1	TxIXA precursor *Conus textile,* Cloth of gold cone	P00608
c85542_g5_i2	TxIXA precursor *Conus textile,* Cloth of gold cone	P00608
c63202_g2_i1	O3 Vc1 prepropeptide *Conus Victoriae*	P06323
c34825_g1_i1	O1 P6.1 precursor *Conus purpurascens*	P03084

c53010_g1_i2	O1 Ar6.9 precursor *Conus arenatus*	P00738
c63916_g1_i1	O2 Vc6.13 precursor *Conus victoriae*	P04274
c63916_g1_i2	O2 Vc6.13 precursor *Conus victoriae*	P04274
c69163_g3_i1	T Pu5.5 precursor *Conus pulicarius*	P02797
c71244_g1_i1	M Conomarphin Bt3 precursor *Conus betulinus*	P05914
c72308_g5_i1	O1 ViKr92 precursor *Conus virgo*	P01129
c72308_g5_i2	O1 ViKr92 precursor *Conus virgo*	P01129
c73929_g1_i1	O1 Ar6.2 precursor *Conus arenatus*	P00725
c73929_g1_i2	O1 Ar6.2 precursor *Conus arenatus*	P00725
c80317_g2_i3	O1 Ml6.2 precursor *Conus miliaris*	P02766
c80317_g2_i5	O1 Ar6.18 precursor *Conus arenatus*	P00747
c82150_g1_i1	O1 Ar6.9 precursor *Conus arenatus*	P00738
c83924_g4_i1	J Bt14.1 precursor *Conus betulinus*	P05891
c86011_g1_i1	O1 Vn6.11 precursor *Conus ventricosus*	P00722
c86011_g1_i4	O1 Vn6.11 precursor *Conus ventricosus*	P00722
c86011_g1_i5	O1 Pu6.2 precursor *Conus pulicarius*	P03133
c119467_g1_i1	M Pu3.6 precursor *Conus pulicarius*	P04208
c39517_g1_i1	M Vt3 Y01 precursor *Conus vitulinus*	P04708
c45646_g1_i1	O1 MiK42 precursor *Conus miles*	P01111
c63916_g1_i3	O2 Vn6.2 precursor *Conus ventricosus*	P00692
c69791_g1_i1	M Conomarphin Bt1 precursor *Conus betulinus*	P05912
c73929_g1_i7	O1 Ca6.1 precursor *Conus caracteristicus*	P03129
c75039_g1_i1	O3 Pu6.37 precursor *Conus pulicarius*	P05636
c75039_g1_i3	O3 Pu6.37 precursor *Conus pulicarius*	P05636
c75039_g1_i6	O3 Pu6.37 precursor *Conus pulicarius*	P05636
c75039_g1_i8	O3 Pu6.37 precursor *Conus pulicarius*	P05636
c75039_g1_i9	O3 Pu6.37 precursor *Conus pulicarius*	P05636
c75788_g1_i1	D VxXXA precursor *Conus vexillum*	P03630
c77403_g4_i1	O1 Pu6.15 precursor *Conus pulicarius*	P05591
c77403_g5_i1	O1 MiK42 precursor *Conus miles*	P01111
c79247_g1_i3	D Cp20.4 precursor *Conus capitaneus*	P03592
c79895_g2_i1	A Pu1.4 precursor *Conus pulicarius*	P02863
c79895_g2_i2	A Pu1.4 precursor *Conus pulicarius*	P02863
c79895_g2_i9	A Pu1.4 precursor *Conus pulicarius*	P02863
c83594_g2_i1	T TsMLCL 02 precursor *Conus tessulatus*	P00684
c83594_g1_i2	T ArMLCL 022 *Conus arenatus*	P00689
c85067_g1_i2	I3 Ca11.3 precursor *Conus caracteristicus*	P03797
c85067_g1_i4	I3 Ca11.3 precursor *Conus caracteristicus*	P03797

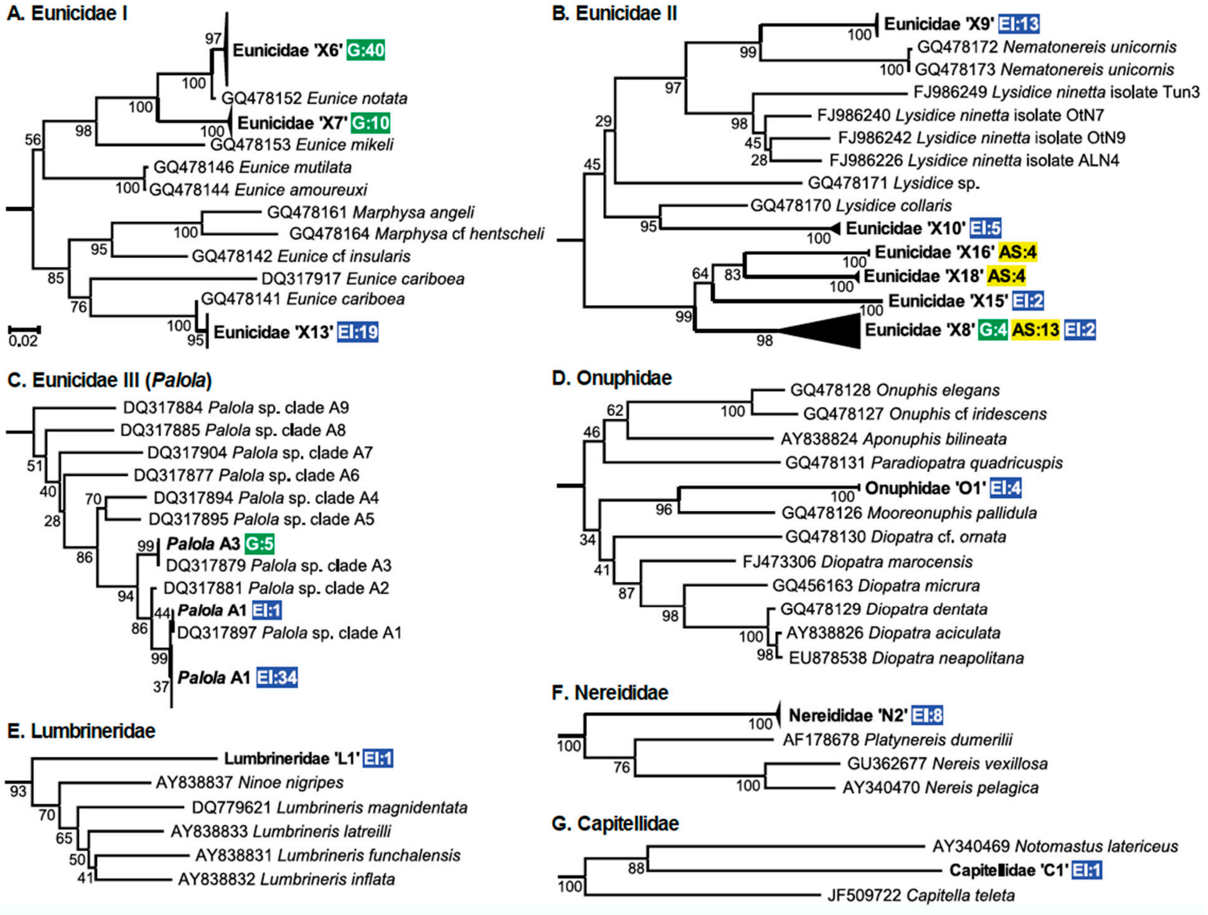

Figure S1. Neighbor-joining trees constructed with Kimura 2-parameter distances from alignments of sequences recovered from feces or regurgitated worms (bold typeface) from Guam (G), American Samoa (AS) and Easter Island (EI) and polychaete sequences from GenBank (regular typeface; accession numbers included in names). Number of sequences of prey recovered from each population is given with population abbreviations next to sequences. Scale bar applies to all trees.
